# SLAE–CPS: Smart Lean Automation Engine Enabled by Cyber-Physical Systems Technologies

**DOI:** 10.3390/s17071500

**Published:** 2017-06-28

**Authors:** Jing Ma, Qiang Wang, Zhibiao Zhao

**Affiliations:** 1 Department of Industrial Engineering, Tsinghua University, Beijing 100084, China; mj_tsinghua_ie@mail.tsinghua.edu.cn; 2 China Norinco Group Planning and Research Institute, Beijing 100053, China; zzbhfut@163.com

**Keywords:** lean automation, Jidoka, lean production, cyber-physical systems, Internet of things, Industry 4.0

## Abstract

In the context of Industry 4.0, the demand for the mass production of highly customized products will lead to complex products and an increasing demand for production system flexibility. Simply implementing lean production-based human-centered production or high automation to improve system flexibility is insufficient. Currently, lean automation (Jidoka) that utilizes cyber-physical systems (CPS) is considered a cost-efficient and effective approach for improving system flexibility under shrinking global economic conditions. Therefore, a smart lean automation engine enabled by CPS technologies (SLAE–CPS), which is based on an analysis of Jidoka functions and the smart capacity of CPS technologies, is proposed in this study to provide an integrated and standardized approach to design and implement a CPS-based smart Jidoka system. A set of comprehensive architecture and standardized key technologies should be presented to achieve the above-mentioned goal. Therefore, a distributed architecture that joins service-oriented architecture, agent, function block (FB), cloud, and Internet of things is proposed to support the flexible configuration, deployment, and performance of SLAE–CPS. Then, several standardized key techniques are proposed under this architecture. The first one is for converting heterogeneous physical data into uniform services for subsequent abnormality analysis and detection. The second one is a set of Jidoka scene rules, which is abstracted based on the analysis of the operator, machine, material, quality, and other factors in different time dimensions. These Jidoka rules can support executive FBs in performing different Jidoka functions. Finally, supported by the integrated and standardized approach of our proposed engine, a case study is conducted to verify the current research results. The proposed SLAE–CPS can serve as an important reference value for combining the benefits of innovative technology and proper methodology.

## 1. Introduction

The market globalization and diversification of customer demands have increased the mass production of highly customized products. Industry 4.0 is the fourth industrial revolution and describes the vision of a smart production system that meets market requirements using information communication technology (ICT), cyber-physical systems (CPS), Internet of things (IoT), and cloud computing [1]. These market requirements will directly lead to complex products and an increasing demand for production system flexibility. However, quality, efficiency, lead times, and cost should be considered when improving production system flexibility under shrinking global economic conditions. Three solutions are now used to improve production system flexibility [2,3,4,5].

(1) Human-centered systems: Among existing production management philosophies and methods, lean production focuses on human-centered production systems [6,7]. Lean production can enable the operators to perform different work tasks, especially assembly tasks. The benefits of lean production, such as waste reduction, human optimization, distributed design, and supply chain management, are well documented and have fully served many companies. However, lean production has the following limitations when applied in Industry 4.0:
  - In traditional lean production, changes in production processes, buffer stocks, or cycle times require laborious adjustments [8]. Therefore, production of complex products requires highly skilled workers who will be trained for a long time and are difficult to find and hire [5].
  - When dealing with complex production tasks, human-centered systems cannot stabilize production and therefore suffer from inconsistent operations, defects, and other problems.
  - Lean production aims to eliminate waste and effectively manage personnel such that each stage of production is done the best way possible. Therefore, lean production eliminates much of the creativity necessary for innovations [3], thereby making enterprises miss advanced technology-push opportunities. Lean production also does not maximize the potential of Industry 4.0 technologies [5].
(2) Automation systems: Automation systems are more stable than human-centered production systems in terms of operation efficiency, quality control, and other factors. Nowadays, robots, automated guided vehicles, automated warehouses, and other high-end automation equipment are widely used in factories to strengthen the said advantages. However, implementing new production systems is a systematic engineering process that integrates all aspects of enterprises. Simply applying high automation technologies to improve production system flexibility will cause problems, such as high investment costs, low investment returns, and investment transformation failure [9,10,11,12]. Although automation technologies are more advanced than human-centered ones, only humans can accomplish some complex and special tasks, especially in assembly operations.
(3) Lean automation: Whether companies can apply lean automation in a shrinking economy while focusing on innovations needed for survival is unclear [3]. Lean automation is emphasized to address this contradictory situation. In the early 1990s, the first approaches for integrating automation technology into lean production arose and were called lean automation. Lean automation is the key pillar of the lean production system and is also called Jidoka or autonomation (collectively called Jidoka hereinafter). The emergence of certain Industry 4.0 technologies (e.g., IoT, CPS, and cloud) has recently widened the application range of Jidoka. Lean production and Industry 4.0 favor decentralized structures over large, complex machines and both aim for small modules with low levels of complexity [8,12]. Therefore, Jidoka is a cost-efficient and effective way to improve production system flexibility in the context of Industry 4.0.

Nowadays, Industry 4.0 technologies and the production flexibility demands can accelerate the development of Jidoka, broaden its contents, and make it smart. Industry 4.0 is propelled by CPS, which constructs a smart system by integrating advanced IoT, embedded control, and cloud computing technologies. This system covers mutual mapping, real-time interaction, and high-efficiency cooperation among man, machine, material, and information at physical and cyber levels; as a result, dynamic allocation of resources and running optimization of the system are enabled [13]. CPS technologies can provide a production system with flexibility, thereby enabling the mass production of highly customized products [14,15].

Although Jidoka has recently gained considerable attention in the context of Industry 4.0 [2,3,4,5,8,10,16,17,18,19,20], the following major research questions still exist:

- CPS-based Jidoka is a hybrid system (physical, conversion, and cyber automation) composed of diverse components, including analog and digital parts, actuators, controllers, ICT, software, and Jidoka rules. CPS-based Jidoka is distributed, such as network-embedded systems that can decouple centralized control and support distributed control. IoT has the power to achieve seamless connections, and each network-connected resource is easily controlled. Cloud technology can support remote and distributed applications. Therefore, a comprehensive architecture should be proposed to support the application of smart Jidoka before CPS-enabled Jidoka is studied. However, this area is yet to be explored.
- Under IoT, networks and protocols are heterogeneous and complex; thus, the data analysis and decision support capability of Jidoka are limited. Therefore, the second stage of CPS-based Jidoka application is converting heterogeneous network or protocols into uniform communication services. This issue is also not addressed by the related studies.
- IoT connects much resource to the production networks, thereby enabling the collection of various states of resources. This function allows Jidoka to detect abnormalities (e.g., operational sequence for operators, material shortage, and buffer capacity). However, existing research incomprehensively present production abnormalities.
- The potential of CPS has led to new fields of application for Jidoka. However, existing approaches are individualized solutions that are tailored to different needs. Thus, modularization and changeability are not supported.

Therefore, a smart lean automation engine enabled by CPS technologies (SLAE-CPS) is proposed based on an analysis of Jidoka and the smart capacity of CPS technologies to address the above-mentioned research issues. This study aims to provide an integrated and standardized approach to design and implement a CPS-based smart Jidoka system. To achieve this goal, a set of comprehensive architecture and standardized key technologies are proposed based on service-oriented architecture (SOA), agent, function block (FB), cloud, and IoT. This CPS-enabled smart lean automation system is composed of sensors, actuators, controllers, ICT, software parts, and Jidoka rules, among others, and can autonomously convert heterogeneous data, analyze data, detect abnormalities, and control feedback. Compared with individualized solutions, SLAE–CPS is smarter and has a comprehensive architecture and standardized key technologies that support standardization, modularization, and changeability.

The remainder of the paper is organized as follows. Section 2.1 gives an overview of the related architecture in the context of Industry 4.0. Section 2.2 describes the capability and typical industrial applications of CPS. In Section 2.3, lean production is introduced and related works on Jidoka are given. Section 3 proposes the architecture of SLAE–CPS; a distributed SLAE–CPS agent and its components and operation principles are also included in this section. In Section 4, SLAE-mediator FB, Jidoka scene rules, and three kinds of executive FBs for the SLAE–CPS agent are presented in detail. Then, a case study is conducted to demonstrate the practicability of the proposed SLAE–CPS. In the last section, conclusions are drawn and the challenges and future research objectives of SLAE–CPS are discussed.

## 2. Related Works

### 2.1. Related Architectures in the Context of Industry 4.0

Architecture is described variously as an inherent aspect of systems or a description of the key characteristics of system composition, functionality, and behavior, and the rules and guidelines governing the design [21]. A system’s architecture differs from its architecture description. Under Industry 4.0, the architecture is the first step to investigate a system. However, the term architecture has various definitions in the context of Industry 4.0. As indicated by our review of the related works, some architectures are conceptual, general, or referential (collectively called general architecture hereinafter); others are specific (e.g., functional and technical). The general architectures aim to provide a guideline for developing and deploying a specific system. The specific systems need detailed architecture and key technologies that have to be tailored to functional needs. Jidoka is a specific system (subsystem) that detects abnormalities and controls feedback under the overall production system. 

This paper discusses four types of general architectures related to production systems: Industry 4.0, IoT, CPS, and cyber-physical production system (CPPS) architectures. The four general architectures can provide some guidelines for developing and deploying a smart Jidoka system. Given the functional difference in specific system architectures, related works only on Jidoka architectures are discussed.

- Industry 4.0 architectures: The Zentralverband Elektrotechnik (ZVEI) of Germany defined the reference architectural model Industry 4.0 (RAMI 4.0) [22], which consisted of a three-dimensional coordinate system that describes all crucial aspects of Industry 4.0. Based on RAMI 4.0, Industry 4.0 technologies can be classified and further developed.
- IoT architectures: Gubbi et al. presented a cloud centric vision for worldwide implementation of IoT [23]. D. Xu et al. defined a four-layered architecture for IoT in industries [24].
- CPS architectures: Lee and Bagheri proposed a five-level CPS structure (smart connection, data-to-information conversion, cyber, cognition, and configuration levels), namely, the 5C architecture, and provided a guideline for developing and deploying a CPS for manufacturing application [13]. Hu et al. presented a generic CPS architecture based on SOA to improve the integration flexibility and process composability of CPS [25]. Lin et al. put forward a semantic agent framework for CPS to enable distributed intelligence and knowledge sharing [26].
- CPPS architectures: Jakovljevic et al. described the IEC-61499 which can be used for modeling a distributed CPPS. The scope of this standard is the reference architecture for utilization of FB for effective system integration in distributed control systems [27]. Pérez et al. proposed a general CPPS architecture, which is composed of production process, information exchange, and plant information models to represent the physical world, the information exchange, and the information to be accessed [28].
- Jidoka architectures: Jidoka is a functional production system that detects abnormalities and controls feedback. Therefore, a simple or incomplete functional architecture cannot support a standardized Jidoka application. As mentioned in Section 1, the existing studies do not provide a comprehensive architecture that presents a hybrid and distributed Jidoka in the context of Industry 4.0. This question has been highlighted in 2015 [8]. Although standardized solutions are preferred to individualized solutions to date, some individualized solutions containing architectures and mechanisms for Jidoka are also significant and are discussed in Section 2.3.

### 2.2. Smart Capacity of CPS Technologies

CPS architectures are the enabling technologies of Industry 4.0 and can make intelligent factories by bringing the virtual and physical worlds together to create a truly connected world in which intelligent objects communicate and interact with one another. CPS in an industrial environment can be regarded as cooperation systems that use cyber technologies embedded in and interacting with physical components that combine IoT, industrial agents, ubiquitous computation, cloud system, and SOA to make factories intelligent [29]. Thus, the above-mentioned technologies are the key technologies of CPS. The smart capacity of CPS technologies is discussed as follows to show how CPS technologies can make Jidoka smart.

- IoT enables CPS by connecting all distributed resources in the industrial environment to gain rich data and a deep knowledge of the environment; accordingly, operators and machines can perform complex tasks with fewer defects. For example, machine vision can be used for quality inspection, assembly verification, and device guidance [30]. For the necessary identification of performed tasks and the individualization of displayed information, the German Research Center for Artificial Intelligence successfully demonstrated a smart manual working station based on augmented reality [8]. Based on the smart manual working station, a continuous flow of pieces can be supported by assisting systems based on augmented reality for employees. Information about cycle times within the visual field of employees supports just-in-time (JIT) manufacturing of goods. In addition, new operators can obtain individualized information about necessary tasks to participate in timed productions. Quick Response (QR) codes, Radio Frequency Identification (RFID), or specific sensors are used to assist operators in identifying a wide variety of materials accurately. Moreover, other kinds of IoT technologies can make operators and machines perform complex tasks with fewer defects.
- Agents are used to achieve distributed intelligence and adaptation for production systems [26,31]. FBs are used to achieve effective component integration in distributed agents [27]. In such distributed and heterogeneous systems, knowledge sharing can be a problem. Therefore, CPS also uses semantics to represent the structure of the shared knowledge, thereby allowing distributed agents to understand themselves during the cooperation processes [26,32,33].
- CPS needs to deal with the problem posed by heterogeneous networks and protocols when integrating components of different natures from different sources. Services can be positioned as higher-level abstractions to allow CPS to mediate the interactions among devices, software, information, humans, and applications within the existing technologies. Currently, SOA has been adopted in various industrial systems due to its integration flexibility and process composability [34,35,36]. Integrating agents and SOAs using agents to embed the intelligent logic control that is exposed as services to the other agents enables the adoption of a unifying technology for all levels of the enterprise [37,38].
- Each engineered system or component must be deployed to support performance. Currently, a large number of resources and services are allowed to deploy to different decentralized locations. Recently, the cloud emerged as a new way to develop and deploy sophisticated applications. Therefore, for supporting centralized and distributed implementations, a complete CPS may be specified as a single model composed of multiple components; the components may be executed or deployed at different locations locally and into the cloud [39]. Cloud-based CPS will be a distributed and intelligent system in the IoT environment [40].

The potential of CPS has facilitated the research and application of designing and integrating CPS into existing production systems [41,42]. For example, Lewandowski et al. developed an architecture for applying CPS in material handling [43]. A Petri net-based approach is proposed in cyber-physical manufacturing systems to support traceability [44]. Kaihara et al. proposed a new approach for CPS-based scheduling and work in process (WIP) control in processing industries [45]. Michniewicz et al. researched the modeling of modular robot cells for the automated planning and execution of assembly tasks based on CPS [46]. However, the deployment of CPS in the industrial environment is still a critical issue that requires proper methodologies that will prepare these systems for industrial usage. The potential of CPS has introduced new applications for Jidoka. However, this issue in the industrial environment remains to be examined [29].

### 2.3. Jidoka: The Application of Automation Technologies to Lean Production

Lean production reduces factors that do not add value to focus on those that do. This management philosophy is derived mostly from the TPS and identified as “lean” only in the 1990s [6,47]. Lean production has two pillars: JIT and Jidoka. JIT means producing only what is needed when it is needed and in the amount needed. JIT is supported by several tools, such as Kanban, Heijunka, and single-minute exchange of die. In the early 1990s, Jidoka began to combine automation technology with lean production. This type of automation implements some supervisory functions rather than production functions. At Toyota, this function stops the machines if an abnormal situation arises and the worker will stop the production line. The abnormality control process consists of four steps: (1) detecting the abnormality; (2) stopping production; (3) fixing or correcting the immediate condition; and (4) investigating the root cause and installing a countermeasure. Jidoka did not gain much attention in the last decade. However, Jidoka is emphasized again due to the rise of Industry 4.0 and is considered a cost-efficient and effective approach for improving system flexibility because of the potential of CPS [2,3,4,5,8,10,16,17,18,19,20]. 

A production system consists of operators, machines, materials, products, the operation environment, and other resources. Therefore, the Jidoka function of abnormality detection is not limited to human–machine interactions, which have mainly been the focus of studies in the last decade. Nowadays, resources can be connected to the production networks, thereby enabling the collection of various states of resources. Therefore, corresponding monitoring–optimization–prediction (MOP) approaches may also be needed to support abnormality detection. MOP approaches are described as follows.

- Individualized solutions for Jidoka: Danielsson et al. described a flexible lean automation concept for a robotized manufacturing industry [48]. Jackson et al. developed a possible Jidoka solution for increased reconfigurability of robotic systems to enable the production of different products, and simple configuration to allow future product variants to be handled without large additional investments [10]. Mashaei and Lennartson investigated the lean design and control of an automated material handling system and introduced a universal framework for modeling and analysis of different types of material handling mechanisms to obtain a minimum number of resources in a system design and fulfill a desired throughput [49]. Similarly, Yamazaki et al. proposed a design method of material handling systems for lean automation; this method focuses on the low utilization rates of mechanisms, such as MUDA, at the equipment level rather than the station level, and devises a method for increasing the utilization rates of the equipment by aggregating the same kind of operations distributed across different stations [18]. Kolberg et al. presented an ongoing work on an interface for digitizing lean production methods using CPS. A model-view-controller-based architecture is proposed for realizing the interface [20]. Karadayi et al. proposed a design methodology for hybrid feeding systems in lean production-based assembly lines [17].
- MOP methods: Individualized Jidoka solutions are supported by related MOP methods. For example, Berk et al. proposed a Jidoka quality control method that combines the conventional use of control charts and randomly occurring system stoppages for inspection or repair decisions [16]. Gao et al. demonstrated an in-process sensing technique for online product quality assessment, and this technique can be used to improve quality detection capability [50]. Królczyk et al. presented a case study of material flow analysis in the production process and proposed a material flow optimization project [51,52]. This project requires creating a program of internal transportation and optimizing the location of the working stands. Aside from monitoring and optimization approaches, prediction technologies are gaining increased attention nowadays. For example, Nadai et al. proposed an equipment failure prediction approach based on neural network analysis incorporated with maintainer inspection findings [53]. A machinery condition prediction based on wavelet and support vector machine can be found in [54]. Jiang et al. designed an early warning model based on time series data for production safety [55]. Dean et al. researched the prediction of manufacturing resource requirements in mass customization production [56]. However, other kinds of MOP approaches can be embedded in the Jidoka system to detect abnormalities based on a comprehensive architecture and standardized key technologies.

Currently, CPS-based Jidoka is considered a cost-efficient and effective approach to improve system flexibility under the shrinking global economic conditions. According to existing studies, CPS can make Jidoka smart using IoT, SOA, agent, FB, semantics, and the cloud. The standardization, modularization, and changeability of Jidoka, which are critical for Industry 4.0, are not supported due to the lack of comprehensive architecture and standardized key technologies. Finally, MOP methods are effective and can be embedded in CPS-based Jidoka systems using of comprehensive architectures and standardized key technologies.

## 3. Definition and Architecture of SLAE–CPS 

**Figure 1:**
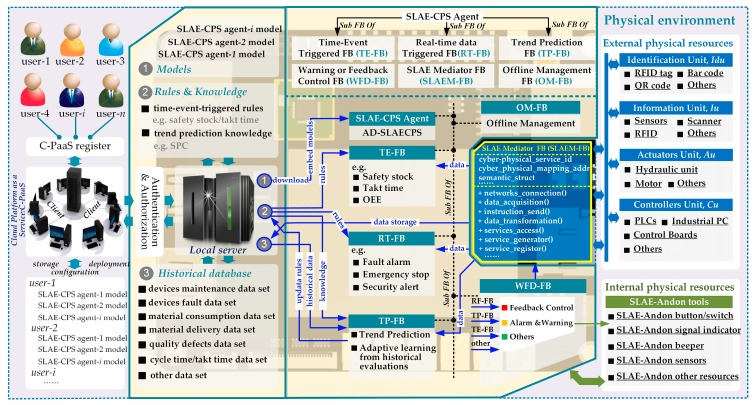
Architecture for CPS-based smart Jidoka engine system.

SLAE–CPS consists of sensors, actuators, controllers, ICT, software parts, and Jidoka rules and can convert heterogeneous data, analyze data, detect abnormalities, and control feedback autonomously. The proposed SLAE–CPS structure is shown in Figure 1. SLAE–CPS is more advantageous than the established Jidoka because the former uses the open and loosely coupled structure of the IoT environment. SLAE–CPS is composed of several SLAE–CPS agents that can accomplish the same functions, operate independently, and achieve decentralization, interconnection, and enhanced monitoring functions with the support of CPS technologies. Moreover, SLAE–CPS is equipped with double deployment: local deployment and cloud-platform as a service (C-PaaS) deployment. C-PaaS can terminate the coupling relationship between the SLAE–CPS application and the physical environment and support the customization configuration and remote cloud deployment of SLAE–CPS scenarios on the C-PaaS platform based on scene element abstract and physical environment virtualization. Therefore, SLAE–CPS possesses high deployability and reconstitution. Scene models are deployed in IoT-embedded systems, mobile terminals, or other workstations by use of a distributed scene actuator as the original performance part of the SLAE–CPS agent. The agent is responsible for downloading SLAE–CPS Jidoka scenes into the local platform and on the C-PaaS platform, thereby responding to the activation indexes of SLAE–CPS scenes under multiple modes (e.g., event driving, real-time data driving, and trend prediction) and executing an alarm or stopping production immediately according to grades of production abnormality. Finally, with respect to optimization methods, the knowledge rule generator of the SLAE–CPS agent is used as the primary learning part of the SLAE–CPS agent that is responsible for the deep statistical analysis and trend prediction on abundant scene running data, mining, and production of new agent running and activation rules on this basis. This process supports new SLAE–CPS scene activation methods and realizes automatic updates to scene activation rules and trend prediction to some extent.

**Figure 2:**
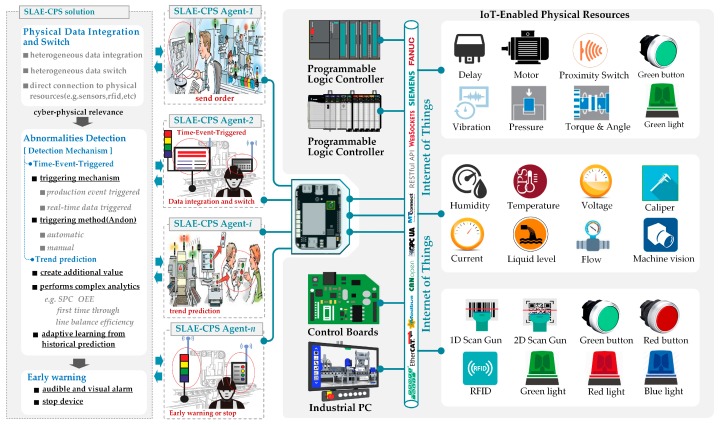
Cyber-Physical environment of CPS-based smart Jidoka engine system.

Figure 1 shows that the entire structure is composed mainly of following parts:

(1) IoT-Enabled Physical Unit (*IoT-Pu*): CPS presents a high combination and coordination between physical and computational elements. In CPS, physical and software components are deeply intertwined. In this study, physical elements are called *IoT-Pu*. In Figure 2, the entire *IoT-Pu* can be classified into Identifying Unit (*Idu*), Information Unit (*Iu*), Actuators Unit (*Au*), and Controllers Unit (*Cu*).
  - *Idu:* This part is responsible for addressing the sensing of normal assembly resources (e.g., Materials, WIP, product, and material-handling devices) by *Iu*. The general approach is to attach some identifying units (e.g., RFID tags, barcodes, GPS, and IC cards) to the assembly resources. These identified resources can be sensed and recognized by some *Iu* (e.g., RFID, scanning guns, and IC machines). Therefore, based on the identified resources, some *Iu* can collect resource data and capture abnormal production data automatically.
  - *Iu***:** This part is responsible for perceiving (collecting) and transmitting production environmental data to *Cu* and activating corresponding SLAE–CPS scenes, such as scanning gun, RFID, machine vision, digital quantity and analog quantity sensor.
  - *Au*: This part is responsible for executing *Cu* control commanders, responding to auto-stop commanders of SLAE–CPS Agent, and implementing corresponding logic actions (e.g., hardware with actual execution functions) or logic control functions (e.g., relay, servo motor, cylinder, and hydraulic equipment).
  - *Cu*: This part is responsible for receiving cyber commands and controlling *Idu*, *Iu*, and *Au* to accomplish production tasks. *Cu* is composed of thing processing units with unified physical distributed logics (e.g., device clusters with independent transmission), data storage, and logical judgment capability (e.g., programmable processor, industrial personal computer, and embedded board card).
(2) SLAE–CPS Agent: The cyber-physical agent integrated with a hardware module and software component can access operating environmental data easily and execute poka-yoke (PY), anomaly detection (AD), trend forecast (TF), feedback (FED), and control and warning function (PY–AD–TF–FED function). An agent with independent communication, operation, and feedback control functions is called a SLAE–CPS agent. As an important component of the SLAE–CPS, the SLAE–CPS agent mainly consists of actuators for distributed SLAE–CPS scenarios (*AD-SLAECPS*) and six FBs with different functions, namely, SLAE-mediator FB (*SLAEM-FB*), real-time data-triggered FB (*RT-FB*), time-event-triggered FB (*TE-FB*), trend prediction FB (*TP-FB*), warning and feedback control FB (*WFD-FB*), and offline management FB (*OM-FB*).
  - *AD-SLAECPS*: As the core part of the SLAE–CPS agent, *AD-SLAECPS* is mainly responsible for downloading SLAE–CPS scene models into the local platform and into the C-PaaS platform and controlling six types of FBs to execute SLAE–CPS scene models under multiple modes (e.g., event driving, real-time data driving, and trend prediction). Meanwhile, *AD-SLAECPS* executes corresponding feedback control (e.g., alarming and production termination) functions according to abnormality grade. According to the practical requirements, the scene models can be configured as services, applications (web or desktop), or other types of executable models (e.g., Petri net model [44,57,58]) based on the requirement of different Jidoka scene parameters (Table 1) for the SLAE–CPS agent. In this study, the scene models are programmed as desktop applications and are introduced in Section 5.
  - *SLAEM-FB*: This FB provides a service-call function that can acquire heterogeneous data, manage the connections among various types of networks through various industrial Ethernet protocols and communication technologies, and uniformize the heterogeneous data. Through this function, *SLAEM-FB* provides uniform CPS access to the SLAE–CPS agent.
  - *RT-FB*: As one of the basic software FBs of the SLAE–CPS agent, *RT-FB* mainly executes the SLAE–CPS scene activation function under the real-time data triggering category. *RT-FB* registers the desired data request services according to scene configuration and triggers the corresponding SLAE–CPS scenes based on real-time data. Examples of these scenes include alarms when operating personnel enter a dangerous operation region, equipment alarms, and Andon alarms (e.g., team Andon, quality defect Andon, equipment exception Andon, and quality defect Andon).
  - *TE-FB*: This FB is one of the basic software FBs of the SLAE–CPS agent and mainly performs SLAE–CPS scene tests and time-event triggering. Embedded time and events in *TE-FB* cannot be triggered by real-time data and can only be triggered when the set threshold is met. Examples of this threshold include safety stocks for preventing store deficiencies, station timers for preventing imbalance in production lines, overall equipment efficiency, employee efficiency, and planned shut-down clocks.
  - *TP-FB*: *TP*-*FB* combines historical running data of the system to analyze and mine potential abnormal factors in the production process that *TE*-*FB* and *RT*-*FB* fail to detect. As a basic software functional block of the SLAE–CPS agent, *TP*-*FB* analyzes abnormalities on the basis of mature data statistical analyses and mining algorithms and by setting knowledge rules to predict possible system anomalies and sending pre-alarms. These pre-alarms include those for quality anomaly decision rules based on statistical process control (SPC), curves based on mass historical data (material delivery timing of different production batches and different products) for the predicted material delivery timing of the current production batch, and for fluctuations in operating personnel’s competency index. *TP*-*FB* can offer predictive device maintenance with a relatively mature failure prediction algorithm. In addition, *TP-FB* is the learning part of the SLAE–CPS agent and can make deep statistical analyses on abundant SLAE–CPS scene running data and produce new engine activation rules, such as statistics, updates in scene word frequency indexes, updates in SPC anomaly decision rules, and updates in competency index evaluation models. *TP*-*FB* can automatically update scene activation rules and trend prediction to some extent.
  - *WFD-FB*: The SLAE–CPS agent is a cyber-physical agent integrated with a hardware module and software component. *WFD*–*FB* is the alarm and feedback control FB of the SLAE–CPS agent. This FB can control the built-in hardware module (e.g., warning light and buzzer) of the SLAE–CPS agent and establish the feedback control communication channel with *IoT-Pu* through *SLAEM-FB*. *WFD-FB* executes alarm and control functions according to abnormality grade after receiving feedback commands from *TE-FB*, *RT-FB*, and *TP-FB*.
  - *OM-FB*: This FB is the knowledge localization and offline data management part of the SLAE–CPS agent. To prevent SLAE–CPS agent failure caused by linkage interruption between the SLAE–CPS agent at the workshop’s upper and lower servers, *OM-FB* automatically updates the desired data and knowledge rules for the operation of the current SLAE–CPS agent regularly or after the SLAE–CPS agent is started. Moreover, *OM*-*FB* can offer offline storage of SLAE–CPS agent running data and transmission of SLAE–CPS agent running data to the server after communication restoration and updating of knowledge rules. As a result, completeness of global data and knowledge rules is ensured.
(3) SLAE Server: This server provides SLAE–CPS agent applications for enterprise knowledge rule storage, historical data storage, and model configuration and storage. Based on the certification of the C-PaaS platform, SLAE–CPS agent application models that are authorized and configured in the C-PaaS platform can be downloaded and updated.
(4) C-PaaS: C-PaaS register functions are needed for SLAE–CPS agent applications for different types of customers and factories. A system supplier can perform SLAE–CPS agent application model configuration and deployment functions on the C-PaaS platform according to customer needs.

## 4. SLAE–CPS Agent

As the core component of SLAE–CPS, the SLAE–CPS agent is mainly composed of six FBs with different functions. *SLAEM-FB* and other FBs are explained in detail below.

### 4.1. SLAEM-FB

**Figure 3:**
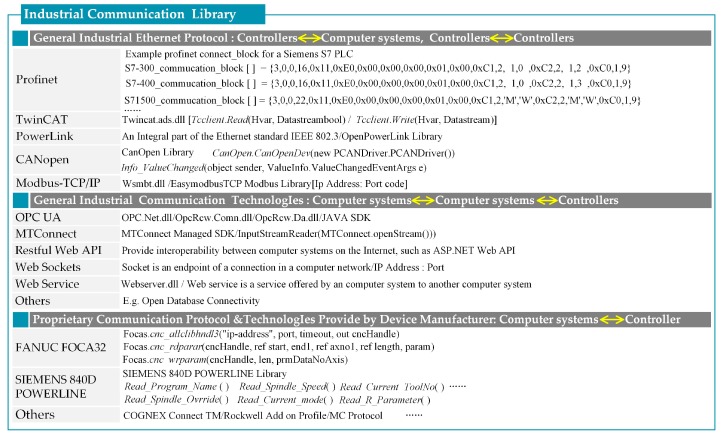
Industrial communication library.

Data in a production system can be collected from heterogeneous sources across different production system levels. These sources include different physical resources (e.g., sensors, actuators, and controllers) and management information systems (MIS) (e.g., manufacturing execution system (MES), enterprise resource planning (ERP), product life cycle management (PLM), and product data management (PDM)). One of the first steps for production system management and control is to obtain heterogeneous data, and then uniformize and understand them. Some common Ethernet protocols (e.g., Profinet, TwinCAT, PowerLink, CANopen, and Modbus TCP/IP), communication technologies (e.g., OPC UA, MTConnect, and web service), and various proprietary protocols (e.g., FANUC FOCA 32 and Siemens 840D PowerLink) are developed to address the above-mentioned issues and integrate the data from heterogeneous hardware and software (Figure 3). However, a protocol is a set of rules for a particular type of communication, and different protocols often describe different aspects of a single or specific communication. Web service is usually used by enterprise service buses (ESB) to solve the coordination problem among different MIS, such as ERP, MES, PLM, and PDM. ESB is an integration infrastructure in the software industry and is realized through service containers distributed across a connected environment. To support SOA, the ESB service containers include all important web service technologies. If many different web service protocols, such as hypertext transport protocol, simple object access protocol (SOAP), and file transfer protocol, are to be used and standardized to a single protocol such as SOAP, the ESB can perform the necessary protocol transformation among the different MIS. This condition shows that ESB is an ideal backbone for implementing SOA at the MIS level. Apart from the interaction among different MIS, a seamless method that can manage the acquisition of data, the connection among various types of networks through various protocols and communication technologies, and the homogenization of heterogeneous data at the physical level is also needed [59]. SLAE–CPS is similar to a production system in that the standardization methods must be sought and implemented to support heterogeneity at the architectural and protocol levels [60]. Only in this way can SLAE–CPS achieve high-level functions, such as complex analytics and adaptive learning from historical evaluations.

According to the three types of communication modes, code development uses two modes: operation system-based device controller driver APIs (OS APIs) and web APIs (Figure 3). Three steps, namely, driver installation, dynamic methods link library referencing, and method call execution, are required to make OS APIs available for desktop applications. OS-based APIs are provided by original equipment manufacturers (OEMs) with limited OS and programming language resources. On the contrary, web APIs are independent of OS type and programming language and can be used in C#, .NET, ASP.NET, Java, JavaScript, Python, PHP, Ruby, Android, and any other code development tool that supports communication over networks. However, not all OEMs can provide web APIs. Therefore, a seamless method for data acquisition and transformation is needed and can result in a large range of mobile and convenient Jidoka applications.

**Figure 4:**
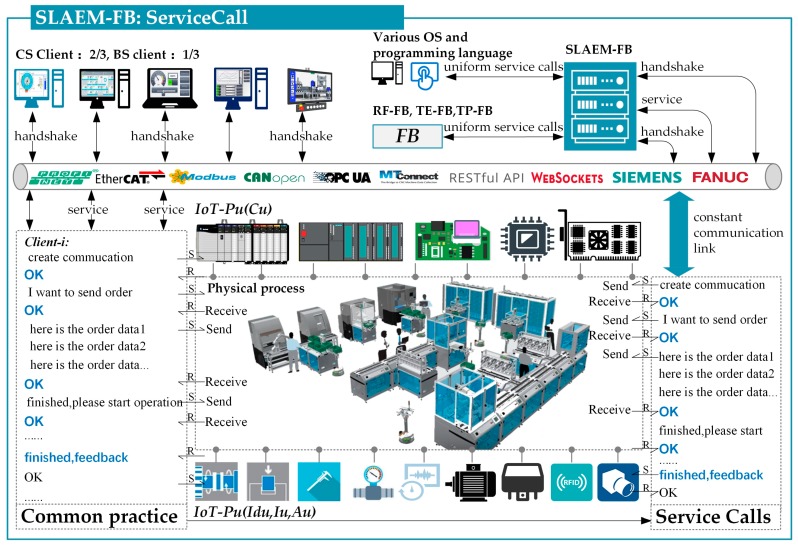
Framework of SLAE-mediator FB.

**Figure 5:**
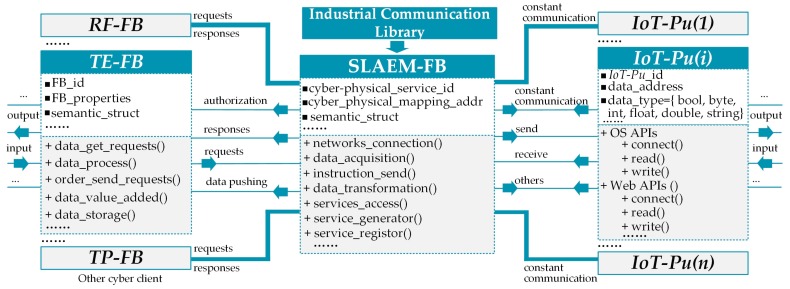
Services-call mechanism.

Therefore, this study proposes a service-oriented *SLAEM-FB* that is independent of OS type and programming language (Figure 4). The *SLAEM-FB* in this study can handle various network connections, heterogeneous data acquisition, and uniform data transformation (switch) at the physical level and provide CPS access to other FBs or even cross-level ESB. The service-call mechanism is shown in Figure 5. *SLAEM-FB* first creates a constant communication link with the *IoT-Pu* base in the communication library (OS APIs and web APIs). Then, a corresponding semantic service will be generated and registered by *AD-SLAECPS*. Each physical address needs to be monitored and added to the semantic service. Finally, according to the real-time requirement of data acquisition and processing in the SLAE–CPS agent application, some data will be pushed to other FBs (*RT-FB*, *TE-FB*, and *TP-FB*) immediately by the *SLAEM–FB*. Meanwhile, each cyber client can send requests to the *SLAEM–FB* and wait for a response. 

*SLAEM-FB* is described as follows:

- *SLAEM-FB* handles various network connections and data acquisition and transformation. Therefore, each cross-platform cyber client does not need to know the physical-level data structure. A cyber client only needs to register and call a corresponding CPS service.
- *SLAEM-FB* can handle real-time updates through constant communication links. Therefore, according to the real-time requirement of data acquisition and processing in practical applications, data request and pushing mode are available.

### 4.2. TE-FB, RT-FB and TP-FB

Based on heterogeneous protocol adaptation and data integration of *SLAEM-FB*, *TE-FB, RT-FB, TP-FB*, and *WFD-FB* accomplish the PY–AD–TF–FED function of the SLAE–CPS agent by use of different types of real-time, time, event, and historical data. Jidoka scene parameters (JDKSPA) or Jidoka rules of PY–AD–TF–FED, which are needed in the SLAE–CPS agent, are analyzed and summarized from operators, equipment, material, and quality (*OEMQ*) in three time dimensions of *RT-FB* (real-time, RT, in Table 1), *RF-TB* (accumulated to triggering threshold of some event and time in some time span, RF, in Table 1), and *TP-FB* (predicted failure factors that may occur in some time period, TP, in Table 1) (Table 1). On the basis of the JDKSPA in Table 1, mature mathematical model and measurement formula are accessed to and embedded in the corresponding *TE-FB, RF-TB*, and *TP-FB*, and the PY–AD–TF–FED function of the SLAE–CPS agent is realized through data driving. Feedbacks of each type of JDKSPA in Table 1 are classified simultaneously. They are mainly divided into control and warning (C and W in Table 1, respectively). This classification is not fixed and can be alternated according to different technologies and production environmental demands.

In Table 1, Jidoka factors = {*Fo*, *Fe*, *Fm*, *Fq*, *Ft*}. *Fo* = {*Fo*_1_, *Fo*_2_, *Fo*_3_, *Fo*_4_, *Fo*_5_, *Fo*_6_, *Fo*_7_, *Fo*_8_, *Fo*_9_}, where *Fo*_7_ measures whether operating process of workers meets different requirements of standard operations. *Fe =* {*Fe*_1_, *Fe*_2_, *Fe*_3_, *Fe*_4_, *Fe*_5_, *Fe*_6_, *Fe*_7_, *Fe*_8_, *Fe*_9_, *Fe*_10_, *Fe*_11_,}, where *Fe*_2_ is the idle time of an equipment in case the parts cannot go downstream, *Fe*_3_ is the idle time of an equipment in case the parts are stopped at the upstream, and *Fe*_10_ and *Fe*_11_ are the functions that can be realized through data processing, feature extraction, comparison, assessment, and forecast. Each stage should be supported by embedding corresponding mature algorithm models. *Fm* = {*Fm*_1_, *Fm*_2_, *Fm*_3_, *Fm*_4_, *Fm*_5_, *Fm*_6_}, where *Fm*_4_ measures the line edge caused by delayed operation of warehouse department or other reasons or deficiencies in stores aside from the operating station. *Fq* = {*Fq*_1_, *Fq*_2_, *Fq*_3_, *Fq*_4_, *Fq*_5_}, where *Fq*_2_ needs continuous samples of trend changes to determine quality trend. Except for *OEMQ*, *Ft*_1_, *Ft*_2_, and *Ft*_3_ measure timeout of operation station. WIP level and cache capacity are used to measure whether cycle time of station exceeds the Takt time of customer demand and whether the excessive time is within the allowable range. Processing capability and efficiency of operating station are denoted by *Ft*_2_ and *Ft*_3_, respectively. As described in Table 1, most JDKSPA are common. Therefore, JDKSPA that are difficult to be understand, such as *Fo*_7_, *Fe*_2_, *Fe*_3_, and *Fm*_4_, are introduced in the following text.

**Figure 6:**
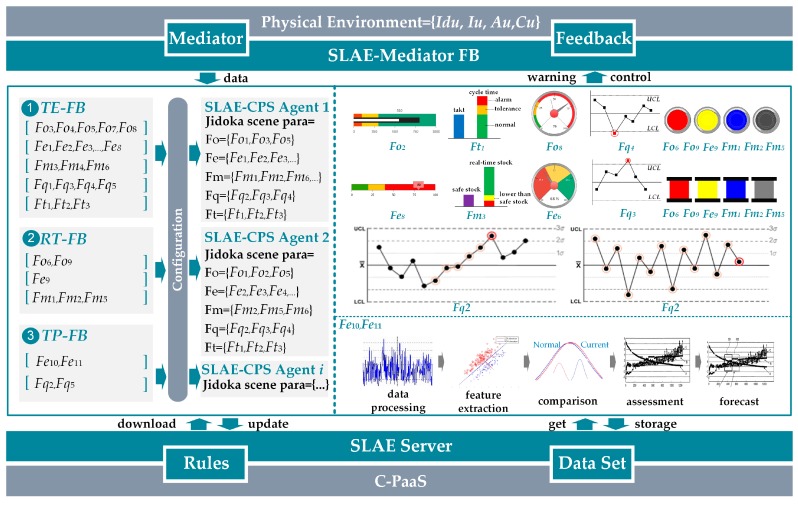
Sample illustrations for *TE-FB*, *RT-TB*, and *TP-FB.*

Classification of JDKSPA according to different time dimensions in Table 1 is shown in Figure 6. Triggering conditions of different JDKSPA are vividly expressed by partial “sample graph.” SLAE–CPS deploys different sets of JDKSPA in response to different demands. As shown in Figure 6, *TERFTP-FB* is equal to cyber-physical shell in the production system which executes Jidoka functions. This FB adopts heterogeneous data of the physical layer through *SLAEM-FB* and converts them into standard independent uniform services driven by protocols and hardware. On this basis, knowledge rules and abundant historical running data are collected from the SLAE server in the cyber layer, and corresponding Jidoka triggering rules are set and embedded into different types of FBs. Accordingly, integration of cyber and physical components is realized and dependency between traditional Jidoka application software and hardware is avoided. Therefore, SLAE–CPS application presents improved distributed characteristics. SLAE–CPS can be deployed either in local area through applications or at C-PaaS end through services.

## 5. Case Study

**Figure 7:**
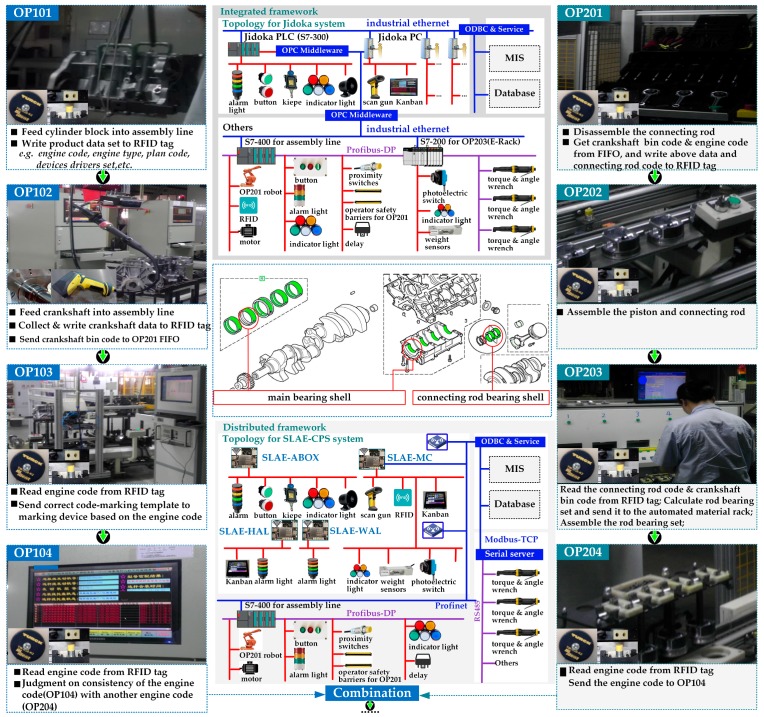
Piston rod assembly region in the engine assembly line.

**Figure 8:**
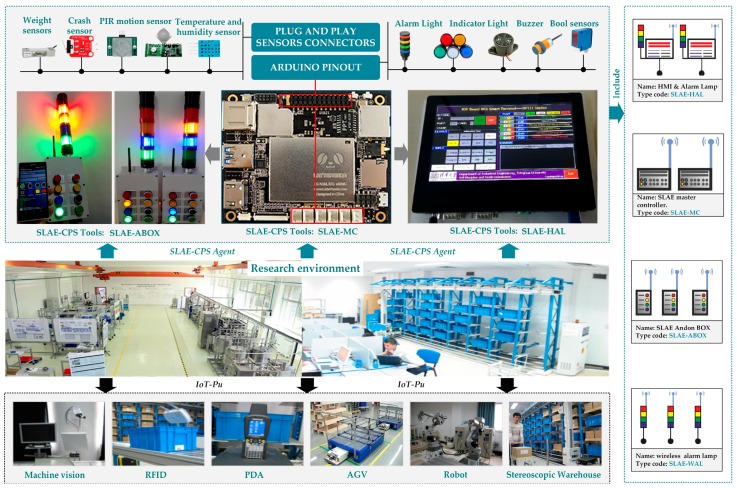
Research environment and results.

Based on the proposed smart lean automation engine, SLAE–CPS tools are developed and tested based on the engine assembly line of an automobile enterprise and Chinese model factory (Figure 7). This Chinese model factory was constructed by McKinsey and the Department of Industrial Engineering of Tsinghua University as a developmental and experimental environment (Figure 8). The entire SLAE–CPS system is composed of hardware module and software component. The hardware module is the secondary development based on LattePanda that can be operated in Windows 10. This module contains a certain quantity of sensor connection pinout and is compatible with Arduino. The software component is web services developed on the .NET platform and human–computer interaction interface.

The developmental and experimental environment is shown in Figure 8. The region with black arrow is the *IoT-Pu* resource in the experimental environment, and the upper region comprises the SLAE–CPS tools integrating hardware modules and software component. According to the SLAE demands of different stations in the production system, the SLAE–CPS tools hardware kit is mainly divided into four classes.

- SLAE Master Controller (SLAE-MC): Only one SLAE-MC is deployed in every production region or sub-production system. This module is used mainly to deploy *AD-SLAECPS* and download and execute the SLAE–CPS configuration scenes, including protocol adaptation, heterogeneous data integration and transmission, and different types of FBs running in the SLAE–CPS agent.
- Human Machine Interface (HMI) and Alarm Lamp (SLAE-HAL): This module receives the command of SLAE-MC and provides visual SLAE functions and human–computer interaction function for important production station.
- SLAE Andon Box (SLAE-ABOX): This module receives the command of SLAE-MC and provides relatively important stations of different types of production indication and anomaly alarm by combining different colors of indicator light and alarm light. Operators can trigger different types of SLAE-ABOX buttons manually according to the abnormality of station to execute corresponding Andon functions.
- Wireless Alarm Lamp (SLAE-WAL): This module receives the command of SLAE-MC and provides the most basic SLAE indication and alarm to stations with few production indications and anomaly alarm.

The piston rod assembly region in the engine assembly line of an automobile enterprise is shown in Figure 7. In the first half assembly of engine, the assembly of connecting rod bearing shell and main bearing shell is the most vulnerable link. The adaptation of main bearing shell depends on the main bearing seat size on the cylinder and main journal size on the bent axle. The adaptation of connecting rod bearing shell depends on the connecting rod diameter on the bent axle and connecting rod diameter on the connecting rod. Detailed technology and information process are shown in Figure 7.

**Figure 9:**
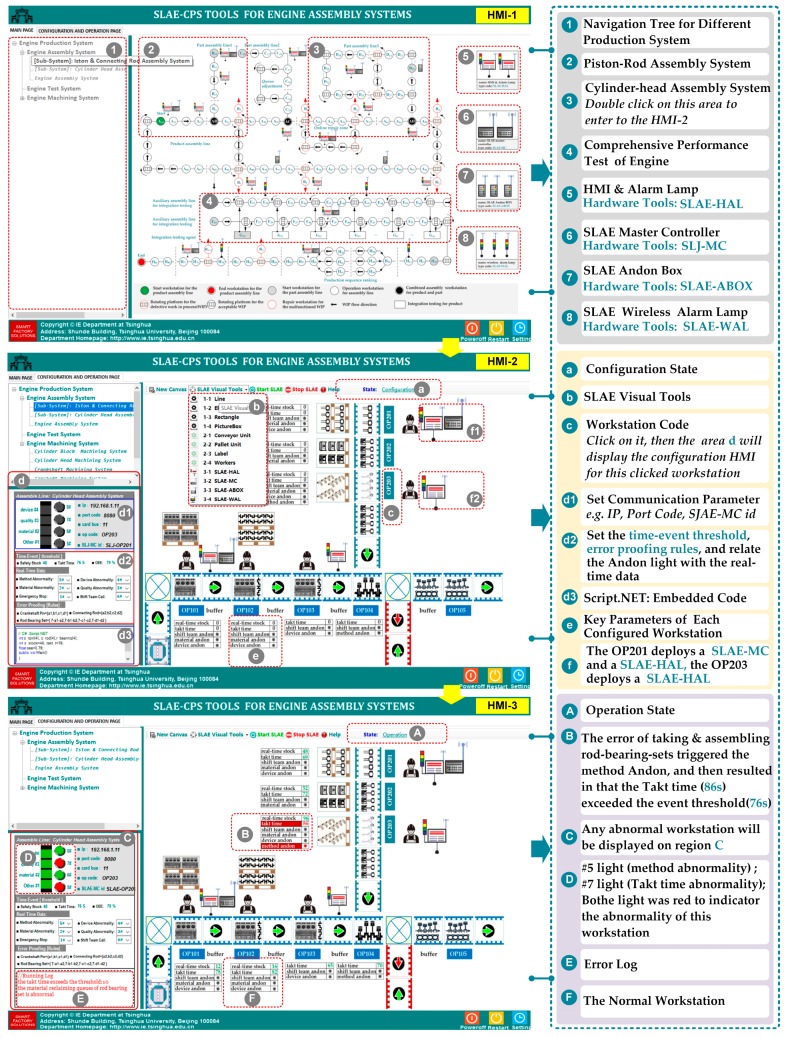
HMI of the SLAE–CPS tools.

This project was implemented in 2012. As shown in Figure 7, in the piston rod assembly region for engine assembly system, some information units (e.g., proximity switches, photoelectric switch, operator safety barriers, weight sensors, torque and angle sensors, and RFID) are connected to one Jidoka PLC; thus, the resulting Jidoka is integrated. In recent years, embedded hardware similar with LattePanda, Arduino, Raspberry PI, and Up-Board are applied in industrial production to support various small distributed application scenes with the rapid development of IoT. With the application of preordered set and technology accumulation, the SLAE–CPS tools (hardware module and software component) that support distributed scene application are developed based on the proposed SLAE–CPS and experimental environment. The SLAE–CPS agent has been verified in laboratory. In the future, the SLAE–CPS tools will be used to update the completed projects and will be applied into new projects. The software component of SLAE–CPS tools is shown in Figure 9 and is mainly responsible for scene configuration of technological process in Figure 8. HMI-1 is the main interface of the system, HMI-2 is the interface for the SLAE–CPS scene configuration, and HMI-3 is the scene operation interface. The software component of SLAE–CPS tools is realized mainly by object-oriented GDI and technology and abstracting and packing desired elements in scene configuration into pixels. In addition, it realizes the desired knowledge rules by embedding various system operation logics and SLAE–CPS in pixels through the Script.NET technology. HMI-1, HMI-2, and HMI-3 are introduced in detail in Figure 9.

The above-mentioned description is combined with the topological structure shown in Figure 7, and the difference (e.g., architecture, communication, flexibility, reliability, and costs) between the integrated Jidoka and distributed SLAE–CPS is introduced (Table 2). Compared with the traditional one, the established SLAE–CPS in this study is more distributed and exhibits better flexibility and reliability. In Figure 7, nearly 50% of resources (e.g., RFID, sensors, buttons, indicator lights, and torque and angle wrench) can be directly connected to decentralized controllers (LattePanda). Thus, only 45% of data (from PLC) need to be transmitted to LattePanda for further processing. Moreover, SLAE–CPS can lead to approximately 50% reduction in total costs.

## 6. Conclusions and Future Works

CPS-based smart Jidoka is considered a cost-efficient and effective approach for improving production system flexibility under a shrinking global economy. SLAE–CPS and its architecture and key technologies are discussed and developed in this study. Compared with individualized solutions, the proposed SLAE–CPS is generally smarter and has more comprehensive architecture and standardized key technologies that support system standardization, changeability, and modularization. The contributions of this study are discussed as follows.

- Compared with individualized solutions, SLAE–CPS presents a comprehensive architecture that joins SOA, agent, FB, cloud, and IoT to support flexible configuration, deployment, and smart performance of Jidoka systems.
- A standardized key technology for converting heterogeneous network or protocols into uniform communication services for a deep and varied abnormality analysis and detection is proposed to strengthen the data analysis and decision support capability of Jidoka.
- In the context of Industry 4.0, IoT can enable a collection of various states of resources. Thus, comprehensive Jidoka scene parameters (or Jidoka rules library) are necessary and useful for a flexible configuration and performance of Jidoka systems.
- Following the comprehensive and standardized approach of SLAE–CPS, a case study is conducted to demonstrate the practicality of the proposed SLAE–CPS. The advantages of SLAE–CPS are further illustrated by a comparison between integrated Jidoka and SLAE–CPS (Table 2).

The advantages of the proposed SLAE–CPS are verified by the case study; however, some challenges, which will be addressed in the future research, still need to be overcome.

- New SLAE–CPS tools based on C-PaaS should be developed to achieve agile implementation and remote data analysis.
- The design should be implemented with security protocols to fully benefit from the many application of CPS. Therefore, the overall security mechanism for SLAE–CPS should be considered, such as sensing security, communication security, actuation control security, and feedback security.
- Although the SLAE–CPS tools have been verified in laboratory, system stability needs to be further tested.
- New mature embedded technologies and human–machine interaction technologies (e.g., augmented reality) should be used to create a smart SLAE–CPS.

## Figures and Tables

**Table 1:** Jidoka scene parameters (Jidoka rules library).

Factors	JDKSPA	FB Type			Feedback	
		RT	TE	TP	C	W
Operator (*Fo_i_*)	*Fo*_1_: Actual personnel work time, *APWT*	☐	☑	☐	☐	☑
	*Fo*_2_: Actual personnel attendance time, *APAT*	☐	☑	☐	☐	☑
	*Fo*_3_: Staff efficiency, *STEF* = *APWT*/*APAT*	☐	☑	☐	☐	☑
	*Fo*_4_: Workstation productivity, *WOPR*	☐	☑	☐	☐	☑
	*Fo*_5_: Workstation passed yield, *WPAY*	☐	☑	☐	☐	☑
	*Fo*_6_: Operational sequence, *OPSE*	☑	☐	☐	☑	☑
	*Fo*_7_: Standard work compliance rate, *SWCR*	☐	☑	☐	☐	☑
	*Fo*_8_: Mis-operation frequency, *MOFR*	☐	☑	☐	☑	☑
	*Fo*_9_: Operator entry in danger zone, *OEDZ*	☑	☐	☐	☑	☑
Equipment (*Fe_i_*)	*Fe*_1_: Equipment downtime, *EQDO*	☐	☑	☐	☐	☑
	*Fe*_2_: Blocking time, *BLTI*	☐	☑	☐	☐	☑
	*Fe*_3_: Starving time, *STTI*	☐	☑	☐	☐	☑
	*Fe*_4_: Available rate, *AVRA*	☐	☑	☐	☐	☑
	*Fe*_5_: Performance Rate, *PERA*	☐	☑	☐	☐	☑
	*Fe*_6_: *OEE* = *AVRA × PERA* × Quality rate (*QURA*)	☐	☑	☐	☐	☑
	*Fe*_7_: Equipment alarm frequency, *EAFR*	☐	☑	☐	☑	☑
	*Fe*_8_: Change over time, *COTI*	☐	☑	☐	☐	☑
	*Fe*_9_: Equipment alarm (real-time), *EQAL*	☑	☐	☐	☑	☑
	*Fe*_10_: Tendency prediction in equipment failure, *TPEF*	☐	☐	☑	☐	☑
	*Fe*_11_: Equipment health forecast, *EQHF*	☐	☐	☑	☐	☑
Material (*Fm_i_*)	*Fm*_1_: Neglected assembly, *NEAS*	☑	☐	☐	☑	☑
	*Fm*_2_: Faulty assembly, *FAAS*	☑	☐	☐	☑	☑
	*Fm*_3_: Safety stock of workstation material, *SSWM*	☐	☑	☐	☐	☑
	*Fm*_4_: Stockout frequency (workstation), *STOF*	☐	☑	☐	☐	☑
	*Fm*_5_: Material shortage (workstation), *MASH*	☑	☐	☐	☑	☑
	*Fm*_6_: Material qualified rate before assembly, *MQRA*	☐	☑	☐	☐	☑
Quality (*Fq_i_*)	*Fq*_1_: *QURA* within workstation	☐	☑	☐	☑	☑
	*Fq*_2_: Quality abnormality pre-warning, *QAPW*	☐	☐	☑	☐	☑
	*Fq*_3_: Exceed upper control limitation of detect, *EUCL*	☐	☑	☐	☑	☑
	*Fq*_4_: Exceed lower control limitation of detect, *ELCL*	☐	☑	☐	☑	☑
	*Fq*_5_: Other quality control rules, *OQCR*	☐	☑	☑	☑	☑
Others (*Ft_i_*)	*Ft*_1_: Job timeout, *JOTI* = Cycle time-Takt time	☐	☑	☐	☐	☑
	*Ft*_2_: Work in process, *WIPR*	☐	☑	☐	☑	☑
	*Ft*_3_: Buffer capacity, *BUCA*	☐	☑	☐	☑	☑

**Table 2:** Difference between integrated Jidoka and SLAE–CPS.

Items	Traditional Jidoka	SLAE–CPS
	**Integrated**	**Distributed**
Architecture	One master controller (PLC). More communication cables. Larger hardware size.	More decentralized controllers. Less (85% reduction in cables). LattePanda: Smaller size.
Data resources	• Data resources: ◎ PLC: 80% data (need transmission). ◎ IPC: 15% data (without). ◎ Others: 5% (need transmission).	• Data resources: ◎ PLC: 45% data (need transmission). ◎ LattePanda: 50% data (without). ◎ Others: 5% (need transmission).
Flexibility	Deployment: local. Deployment speed: longer time. PLC: cannot be controlled remotely. Re-configuration ability: poor.	Deployment: local and C-PaaS. Deployment speed: shorter time. Allowed (Node-RED and Azure IoT). Re-configuration ability: better.
Reliability	One master controller failures will disable all Jidoka functions. Longer fault recovery time: ◎ ≥2 h.	SLAE–CPS controller failures only can disable itself functions. Shorter fault recovery time: ◎ ≤0.5 h.
Cost	Jidoka hardware costs (project in 2012): 70 Andon boxes (contains PLC). 15 PCs. Others (e.g., communication cables). Total costs: 380 thousand RMB.	SLAE–CPS hardware costs: A SLAE–CPS box may cost 3000 RMB. 70 SLAE–CPS boxes need almost 210 thousand RMB. Nearly 50% reduction in total costs.
